# Cardiac function in relation to rhythm outcome after intraoperative epicardial left atrial cryoablation

**DOI:** 10.3109/14017431.2011.592855

**Published:** 2011-08-05

**Authors:** Birgitta Johansson, Birgitta Houltz, Nils Edvardsson, Henrik Scherstén, Thomas Karlsson, Birger Wandt, Eva Berglin

**Affiliations:** 1 Department of Cardiology, Sahlgrenska University Hospital, Gothenburg, Sweden; 2 Department of Clinical Physiology, Sahlgrenska University Hospital, Gothenburg, Sweden; 3 Department of Cardiothoracic Surgery, Sahlgrenska University Hospital, Gothenburg, Sweden; 4 Sahlgrenska Academy at Sahlgrenska University Hospital, Gothenburg, Sweden; 5 Department of Research and Development, Örebro University Hospital, Örebro, Sweden

**Keywords:** atrial fibrillation, echocardiography, intraoperative ablation

## Abstract

**Objectives:**

To assess the effects of intraoperative left atrial epicardial cryoablation on rhythm and atrial and ventricular function.

**Design:**

Thirty five patients with coronary artery disease and documented atrial fibrillation underwent coronary artery bypass surgery and concomitant cryoablation. An age and gender matched control group of 35 patients with atrial fibrillation underwent bypass surgery alone. Echocardiography was performed 9 ± 32 days before and 22 ± 6 months after surgery.

**Results:**

The proportion of patients in sinus rhythm at follow-up was 63% and 34% (p = 0.04) in the cryoablation and control groups, respectively. In patients with sinus rhythm both before surgery and at follow-up, the left atrial area increased (p = 0.002) and the mitral annular excursion during atrial contraction decreased (p = 0.01) after cryoablation. The mitral flow velocity during atrial systole decreased after cryoablation (p = 0.002). The LV diameter increased (p = 0.03) and the left ventricular ejection fraction (LVEF) decreased (p = 0.03) in cryoablated but not in control patients. Continued deterioration was seen in patients with atrial fibrillation both pre- and postoperatively.

**Conclusions:**

At long-term follow-up, a significantly higher proportion of patients was in sinus rhythm in the cryoablation than in the control group. The atrial and ventricular function had decreased at follow-up two years after surgery. This decrease was small and occurred within or close to the reference values in patients with sinus rhythm at follow-up, while patients remaining in atrial fibrillation showed a significant continued deterioration. Some subgroups were small, and the findings, although statistically significant, should be interpreted with caution.

Persistent atrial fibrillation (AF) causes first electrical and then structural remodeling of the myocardium (1). Reversal may occur when the ventricular rate is decreased and regularized during permanent pacemaker treatment following transvenous atrioventricular junction ablation (2). After curative treatment using radiofrequency catheter ablation, an improvement in left ventricular ejection fraction (LVEF) was seen in patients in whom the decreased LVEF was believed to be due to AF *per se* and not primarily to comorbidities (3).

Intraoperative ablation of atrial fibrillation has yielded promising results regarding restoration and maintenance of sinus rhythm (SR) (4,5). Considering that such patients already have advanced underlying structural heart disease as the indication for surgery, the effects on rhythm and other outcomes would not necessarily be the same as in patients primarily treated for AF. Nevertheless, we reported a 62% freedom of symptomatic AF recurrence 32 ± 11 months after radiofrequency current ablation (RFCA) during coronary artery bypass grafting (CABG) and an improvement of health-related quality of life in patients with long-lasting SR (6).

The aim of the present study was to assess the effects on rhythm and atrial and ventricular function after intraoperative left atrial cryoablation.

## Material and methods

### Patients

Patients on the waiting list for CABG and with documented paroxysmal or persistent/permanent AF were eligible for participation in the study. Important exclusion criteria were significant valvular heart disease, hypertrophic cardiomyopathy, permanent pacemaker treatment and previous cardiac surgery. For each patient included, another was matched for age and gender and included as a control patient and underwent CABG but not ablation. First all cryoablation + CABG patients were included on a consecutive basis, thereafter consecutive control patients matched in an age and gender based manner pair-wise on an individual basis. No patient refused to participate, but some were not included because there was no match against a cryoablation patient or because matching patients had already been found and included. All patients were recruited between April 2002 and November 2004. The study was performed in accordance with the Helsinki Declaration of 1975. Written informed consent was obtained from each patient and the study protocol was approved by the Ethics Committee of Sahlgrenska University Hospital (Dnr S 096-02).

### Pre- and postoperative echocardiographic assessment

**Figure 1:**
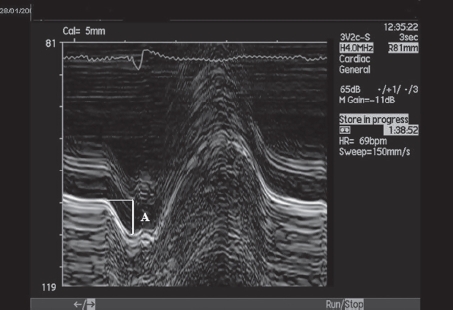
M-mode registration of mitral annulus motion. Atrial contraction (A) is marked. During atrial systole, the left atrial maximal longitudinal axis excursion was measured as the maximum mitral motion during the left atrial contraction.

An echocardiographic examination (7) including tests of the atrial and ventricular function was performed before and two years after surgery. The maximal areas of the atria were measured by planimetry in the apical four-chamber view, excluding pulmonary veins and the left atrial appendage. The left atrial (LA) function was assessed during SR by M-mode recording of mitral ring motion (8,9). The mitral annular excursion during atrial contraction was measured as the maximum mitral motion during the LA systole (Figure 1). The maximal LA ascending excursions were recorded at the septal, lateral, posterior and anterior portion of the mitral annulus, reflecting the regional longitudinal maximal axis contraction excursions, and a mean value was obtained from the four sites. The measurement of the total AV plane displacement has a high reproducibility (10,11).

In addition, the mitral flow velocity during atrial systole was recorded by the pulsed Doppler technique from the apical four-chamber view, with the sample volume located at the tip of the mitral leaflets (12). Left ventricular ejection fraction was evaluated visually in four-chamber and, when available, two-chamber views (8,13) independently by two experienced echocardiographists (BH and a technician), blinded to the identity of the patient. A consensus was reached in case of disagreement. LVEF was also calculated using Simpson's rule in 25 patients. The correlation between the calculated and the visual assessed LVEF was very good (R2 = 0.75). All echocardiographic data were averaged over at least five consecutive beats during AF and three beats during SR. All Doppler data were averaged over at least five consecutive beats regardless of rhythm. All echocardiographic recordings were performed by an experienced echocardiographist (BH) and then stored and analyzed separately by two operators. All of them were blinded to the treatment groups. All analyses were supervised and overread by BH.

### Cryoablation

All operations were performed by the same thoracic surgeons (EB, HS). The cryosurgical probe (Surgifrost 60 mm, ATS CryoMaze Ablation System, ATS Medical Inc., Minneapolis, USA) had a variable freezing segment (4-60 mm long) and an integrated thermocouple for temperature monitoring, capable of reaching a temperature of -160°C using an argon-based cooling system. Cryoablation lesions were applied in an overlapping fashion over a period of 90 s epicardially at each site during cardiopulmonary bypass on the beating and vacuum-emptied heart before aortic cross-clamp and cardioplegic arrest. The lesion set (6) consisted of circles around the right- and left-sided pulmonary veins with a connecting line between the circles. Additional lines were placed from the superior part of the left circle to the top of the left atrial appendage (LAA) and from the inferior part of the left circle over the AV groove fat pad, covering the coronary sinus and corresponding to the P2-P3 section of the mitral valve annulus on the inside. The LAA was closed from the outside with a purse string suture in the cryoablated patients.

### Postoperative management and follow-up

Patients were monitored by telemetry until hospital discharge and received warfarin according to the ACC/AHA/ESC guidelines at the time when the study was performed (14). In the case of recurrent AF, at least one DC cardioversion was attempted. A 12-lead electrocardiogram (ECG) was obtained at three, six and 12 months and at long-term follow-up, and the patients’ current medication, arrhythmic events, DC cardioversions and hospital admissions were reviewed. The long-term follow-up visit included a transthoracic echocardiography. Ablated patients were given amiodarone or sotalol for at least three months and control patients received amiodarone or sotalol as needed.

### Statistical analysis

No statistical calculation was done before the start of study. Based on previous experience with intraoperative RF ablation we assumed that 30 evaluable patients per group would allow for detection of important differences in the restoration and maintenance of sinus rhythm between the groups. Data are presented as mean ± S.D. or percentages unless otherwise stated. Fisher's exact test was used for dichotomous variables for comparisons between groups and the Mann-Whitney U-test was used for continuous/ordered variables. Wilcoxon's signed rank test was used for paired comparisons of changes between time points. A two-step strategy was applied to identify predictors of SR at long-term follow-up. Nineteen baseline factors were first tested univariately for their association with success at long-term follow-up. These factors were age, sex, body mass index, smoking habits, previous hypertension, diabetes, stroke/transient ischemic attack (TIA), myocardial infarction, hyperlipidemia and chronic obstructive pulmonary disease, type and duration of AF, rhythm before surgery, treatment with angiotensin-converting enzyme (ACE) inhibitors, lipid lowering drugs and antiarrhythmic agents at index hospital discharge, left and right atrial area and LVEF. Of these 19 factors, those with a univariate p < 0.30 were tested in a second step for inclusion in a logistic regression model (forward stepwise selection, p < 0.30 for entering and p < 0.05 for staying in the model). The log-rank test was used to test for differences between the cryoablation group and the control group regarding freedom from documented AF/atrial flutter during follow-up, from three months after the operation and onwards. The corresponding Kaplan-Meier curves were calculated. All p-values are two-tailed and considered significant if below 0.05.

## Results

Pre- and peroperative patient characteristics are shown in Table I. The additional extracorporeal circulation time was 21 ± 5 minutes in cryoablated patients. Transthoracic echocardiography was performed 9 ± 32 days prior to and 22 ± 6 months after surgery. At discharge, all patients (except one in the control group) were on antithrombotic treatment: warfarin (n = 45), heparin (n = 30), low molecular weight heparin (n = 2) or aspirin (n = 28). The postoperative rhythm treatment consisted of amiodarone (n = 33 vs. 1), sotalol (n = 9 vs. 6), betablockers (n = 11 vs. 24) and calcium-channel blockers (n = 1 vs. 3) in the ablated and the control patients, respectively. Rhythm medication at follow-up differed little from that before surgery, i.e. amiodarone two versus one, sotalol three versus four, digoxin four versus five and betablockers 22 versus 25 patients in the cryoablation and control groups, respectively.

**Table I:** Patient characteristics.

	Cryoablation + CABG (n = 35)	CABG alone (n = 35)	p#
Age; years	71.7 ± 5.3 (54-81)	70.1 ± 6.8 (53-84)	0.36
Sex; male/female (n)	32/3	32/3	1.00
Body mass index	27.2 ± 3.3 (22.7-36.5)	28.3 ± 3.2 (20.7-38.0)	0.04
Sinus/AF (n)	17/18	11/24	0.22
Type of AF			
Paroxysmal/persistent	20 (57)	21 (60)	1.00
AF history; months (0/1)*	114 ± 112 (11-353)	59 ± 76 (4-264)	0.04
Permanent	15 (43)	14 (40)	1.00
AF history; months (0/3)	103 ± 53 (17-208)	139 ± 91 (16-299)	0.36
Other comorbitidy			
Hypertension	21 (60)	23 (66)	0.80
Diabetes mellitus	10 (29)	11 (31)	1.00
History of embolic stroke/TIA	8 (23)	1 (3)	0.03
Myocardial infarction	24 (69)	16 (46)	0.09
Hyperlipidemia	26 (74)	26 (74)	1.00
Chronic obstructive pulmonary disease	2(6)	1 (3)	1.00
Heart failure	4(11)	3 (9)	1.00
PCI-procedures	4(11)	7 (20)	0.51
Symptomatology			
NYHA functional class (2/0)			0.50**
I	13 (39)	18 (51)	
II	14 (42)	10 (29)	
IIIA	4(12)	5 (14)	
IIIB	2(6)	2(6)	
LVEF; % (4/3)	54.1 ± 11.0 (25-70)	52.8 ± 7.8 (38-70)	0.28
Left atrial area; cm^2^ (4/3)	25.4 ± 5.8 (17-38)	28.0 ± 6.4 (18-48)	0.10
Right atrial area; cm^2^ (4/3)	20.5 ± 5.5 (11-37)	23.1 ± 5.9 (14-45)	0.08
Medication			
Betablocker	25 (71)	29 (83)	0.39
Calcium channel blocker	0(0)	2(6)	0.49
Digoxin	9(26)	6(17)	0.56
Sotalol	5(14)	1 (3)	0.20
Amiodarone	2(6)	0(0)	0.49
ACE-inhibitors/ARB	16 (46)	20 (57)	0.47
Lipid lowering drugs	21 (60)	23 (66)	0.80
Diuretics	12 (34)	15 (43)	0.62
Warfarin/LMWH	20 (57)	19 (54)	1.00
Aspirin/Clopidogrel	24 (69)	20 (57)	0.46

ACE, angiotensin converting enzyme; AF, atrial fibrillation; ARB, angiotensin receptor blocker; CABG, coronary artery bypass grafting;

DC, direct current; LMWH, low molecular weight heparin; LVEF, left ventricular ejection fraction; NYHA, New York Heart Association;

PCI, percutaneous coronary intervention; TIA, transient ischemic attack.

All results are presented as n (%) or mean ± sd (min-max), unless otherwise stated, n = number.

* Number of patients with missing information in the two treatment groups, respectively.

** Ordered degree of functional class used in p-values calculation.

# For difference between treatment groups.

### Rhythm follow-up

**Figure 2:**
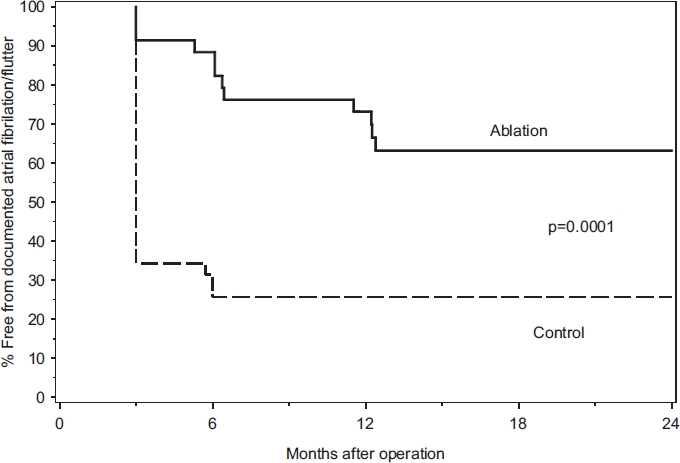
Kaplan-Meier curve showing the freedom from documented atrial fibrillation after the three month therapy stabilization period.

The cumulative freedom from AF or atrial flutter was significantly higher in ablated than in control patients, p = 0.0001 (Figure 2). The proportion of patients in SR in the ablation group versus the control group at three months was 91% vs. 34% (p < 0.0001) and at long-term follow-up 63% vs. 34% (p = 0.04). SR at three months was predictive of that at long-term follow-up (sensitivity 95%, positive predictive value 67%). The multivariate analysis identified SR at the time of surgery and the LA area as predictors of long-term SR.

### Morbidity and mortality during follow-up

A pacemaker was implanted in one patient in the ablation group because of sick sinus syndrome. One patient with CHADS_2_ score 2 developed a TIA after a successful DC cardioversion even though he was on warfarin, and another patient with CHADS_2_ score 0 with SR at follow-up and on aspirin had a stroke with transient visual loss. In the control group, two patients received an ICD six and 13 months postoperatively, respectively, because of recurrent, rapid, monomorphic ventricular tachycardia. Another two patients with CHADS_2_ scores of 1 and 3, the first patient in AF and the second in SR, developed embolic stroke with right-sided paralysis at three months and one month after surgery, respectively. Both were on aspirin because of contraindications for warfarin. Six patients died of reasons unrelated to surgery, ablation or AF.

### Echocardiographic data in the cryoablation and control groups irrespective of rhythm

Comparison between cryoablation and control groups pre- and postoperatively showed a slightly larger RA area (p = 0.04) in the control patients at follow-up (Table II). Regardless of the rhythm at surgery, the LVEF decreased, and the area of both atria increased in both cryo-ablated and control patients, with no statistical difference in the changes between the groups.

**Table II:** Patients before surgery and at follow-up 22 ± 6 months postoperatively (all echocardiographic data included in the results, only patients with echo at corresponding time points included).

	Patients before surgery			Patients at follow-up		
		
	Cryo	Controls	P	Cryo	Controls	P
Males/Females; n	30/3	29/3	1.00	25/3	28/3	1.00
Age (years)	72 ± 5	70 ± 7	0.38	71 ± 5	70 ± 7	0.57
AF paroxysmal/permanent/persistent#; n	18/15/0	15/13/4	0.14	16/12/0	16/11/4	0.19
AF duration# (years) (0/4/0/3)*	10 ± 8	8 ± 8	0.12	9 ± 8	7 ± 8	0.13
median (min-max)	6 (1-29)	3 (< 1-25)		6 (1-29)	3 (< 1-25)	
Sinus/AF or AFL	15/18	11/21	0.45	18/10	11/20	0.04
Heart rate (bpm) (1/0/0/1)*	70 ± 13	71 ± 15	0.81	71 ± 16	67 ± 15	0.33
LV diameter (mm) (6/3/0/1)*	48 ± 5	51 ± 6	0.06	50 ± 6	51 ± 7	0.48
LVEF (%) (2/0/3/1)*	54 ± 11	53 ± 8	0.28	50 ± 10	46 ± 11	0.12
Left atrial area (cm^2^) (2/0/0/0)*	25 ± 6	28 ± 6	0.10	29 ± 7	30 ± 6	0.35
Right atrial area (cm^2^) (2/0/2/1)*	21 ± 5	23 ± 6	0.08	24 ± 6	26 ± 6	0.04

All results are presented as mean ± S.D., unless otherwise stated, n, number; bpm, beats per minute.

* Number of patients where information was missing in the four groups, respectively.

# at baseline.

### Echocardiographic data in patients with sinus rhythm at surgery and at follow-up

Twelve ablation and nine control patients were in SR before surgery and at long-term follow-up, all with a history of paroxysmal AF (Table III). At baseline, the left and right atrial areas were normal and highly significantly smaller than patients with AF on both occasions. In addition to significant increases in the left and right atrial areas, the mitral annular excursion during atrial contraction decreased significantly in cryoablated patients (23% decrease in mean value, p = 0.01). The mitral flow velocity during atrial systole, measured as the A wave on pulsed Doppler recordings, also decreased in the ablation group (33%, p = 0.002). The ablated patients demonstrated a significant increase in the mean value of the LV diameter (10%, p = 0.03) as well, and a decrease in LVEF (6%, p = 0.03). The only significant change in the control group was an increase in the right atrial area.

**Table III:** Patients with sinus rhythm both before surgery and at follow-up 22 ± 6 months postoperatively (only patients with echo both before surgery and at follow-up included).

	Cryo patients (n = 12)			Control patients (n = 9)			
			
	Before surgery	At follow-up	P#	Before surgery	At follow-up	P#	p##
Age (years)	72 ± 6			70 ± 8			
AF paroxysmal/permanent/persistent; n	12/0/0			9/0/0			
AF duration (years)	9 ± 8			7 + 9			
median (min-max)	6 (1-24)			3 (< 1-22)			
Heart rate (bpm) (1/0)*	60 ± 8	63 ± 10	0.43	65 ± 13	59 ± 9	0.07	0.04
LV diameter (mm) (1/1)*	45 ± 5	49 ± 5	0.03	51 ± 7	50 ± 5	0.78	0.04
LVEF (%) (2/0)'	59 ± 6	56 ± 8	0.03	55 ± 9	53 ± 8	0.50	0.61
Left atrial area (cm^2^)	21 ± 4	26 ± 5	0.002	24 ± 4	26 ± 5	0.06	0.28
Right atrial area (cm^2^)	18±4	21 ±4	0.01	19 ±3	23 ±4	0.02	0.99
Mitral flow velocity during atrial systole (m/s) (1/0)*	0.76 ± 0.14	0.51 ± 0.20	0.002	0.70 ± 0.21	0.64 ± 0.26	0.27	0.009
Mitral annular excursion during atrial contraction (cm) (2/0)*	0.60 ± 0.18	0.46 ± 0.15	0.01	0.50 ± 0.14	0.49 ± 0.18	0.65	0.11

All results are presented as mean ± S.D., unless otherwise stated, n, number; bpm, beats per minute.

* Number of patients where information was missing in the cryo and control groups, respectively.

# For change within group

## for difference in change between cryo and control groups.

### Echocardiographic data in patients with atrial fibrillation at surgery and at follow-up

Nine cryoablation and 17 control patients were in AF both before surgery and at follow-up (Table IV), 23 of whom had a history of persistent/permanent AF. In both groups the left and right atrial areas were highly significantly increased at baseline compared to patients with SR on both occasions. The mean value of the left atrial area increased significantly in both cryoablated and control patients (10%, p = 0.047, and 8%, p = 0.03, respectively), while the right atrial area increased in the control patients only (13%, p = 0.0006).The mean LVEF value decreased significantly in the control patients (16%, p = 0.002) but not in the cryoablated group. There were no statistically significant differences in the changes between the cryoablation and control patients.

**Table IV:** Patients with atrial fibrillation/flutter both before surgery and at follow-up 22 ± 6 months postoperatively (only patients with echo both before surgery and at follow-up included).

	Cryo patients (n = 9)			Control patients (n = 17)			
			
	Before surgery	At follow-up	P#	Before surgery	At follow-up	P#	P##
Age (years)	70 ± 7			70 ± 6			
AF paroxysmal/permanent/persistent; n	0/9/0			3/11/3			
AF duration (years) (0/3)*	8 ± 5			9 ± 8			
median (min-max)	8 (1-17)			6 (< 1-25)			
Heart rate (bpm)	76 ± 8	83 ± 18	0.34	72 ± 13	74 ± 16	0.81	0.45
LV diameter (mm) (1/2)*	51 ±4	49 ± 6	0.19	51 ± 6	51 ± 6	0.81	0.28
LVEF (%)	47 ± 15	45 ± 11	0.47	53 ± 8	44 + 9	0.002	0.21
Left atrial area (cm^2^) (1/0)*	30 ± 7	33 ± 8	0.047	30 ± 7	33 ± 5	0.03	0.94
Right atrial area (cm^2^) (2/0)*	24 ±4	28 ± 7	0.09	26 ± 6	29 ± 6	0.0006	0.96

All results are presented as mean ± S.D., unless otherwise stated, n, number; bpm, beats per minute.

* number of patients where information was missing in the cryo and control groups, respectively.

# for change within group

## for difference in change between cryo and control groups.

## Discussion

Significantly more patients, who had undergone CABG and cryoablation were in SR at long-term follow-up as compared to those after CABG alone. Left and right atrial and left ventricular function deteriorated after versus before surgery regardless of whether the patients had been cryoablated. Cryoablated patients in SR both before surgery and at long-term follow-up had normal left and right atrial areas at baseline and showed an increase at follow-up with a decreased left atrial function expressed as a low mitral flow velocity during atrial systole and a low mitral annular excursion during atrial contraction. Most changes were small and occurred within or close to the normal ranges of the variables. While the changes in LVEF in patients in SR before and after intervention may not be significant, considering the precision of the method used, the change in control patients with AF before and after intervention were large enough to represent a true deterioration. Patients with AF at surgery and at long-term follow-up had enlarged left and right atrial areas preoperatively, and there was further enlargement in the control patients.

All patients had significant chronic ischemic heart disease and many had had previous myocardial infarctions. One possible explanation for the decreased cardiac function in spite of that revascularization was performed in all patients, might be that the structural remodeling caused by long-term exposure to AF led to a decrease in ventricular function that made patients more vulnerable to continued deterioration in case of continued AF, as well as less prone to recovery in those restoring and maintaining SR. This hypothesis is somewhat supported by that cryoablated patients in AF at both operation and follow-up had lower LVEF, larger LV diameter and larger left and right atria at baseline than those in whom SR was restored and maintained.

The surgical procedure was the same with the exception of the ablation and the exclusion of the left atrial appendage. Any difference between patients in SR and AF on both occasions would most likely be real and provide additional proof of the vicious circle of continuing AF as a promotor of deterioration of atrial function and dimensions (15). Assessment of atrial dimensions and function has implications in predicting long-term SR and the risk of AF recurrence. Significant reverse remodeling of left atrial, and diastolic and systolic left ventricular diameters was seen in association with a 65% freedom from AF recurrences 30 months after endocardial RF ablation in patients with persistent/permanent AF undergoing mitral valve surgery (MVS) (4). In a study by Reyes et al. (16) intraoperative cryoablation of chronic AF in combination with mitral valve surgery resulted in SR in 82% of the patients at six months. Sinus rhythm was associated with hemodynamically effective contractions on Doppler recordings of the transmitral flow in 70% and of the transtricuspidal flow in 100%. Similar echocardiographic results at 10 year follow-up were reported by Sueda et al. (17) in patients with chronic AF undergoing mitral valve surgery and a simple left atrial procedure. The exact mechanism to explain why 30-40% of their patients did not have effective left atrial contractions was unclear. In contrast none of the patients in SR in the present study showed lack of atrial function.

Our observations are not completely new, and other groups have described compromised atrial function after intraoperative ablation.Yuda et al. (18) described a reduced left atrial (LA) filling fraction 12 months after MVS combined with modified maze surgery for chronic atrial fibrillation as well as after modified maze surgery alone. The authors proposed that the incisions of the LA wall and excision of the LA appendage in combination with non-physiological coupling of atrioventricular contractions caused by delayed LA activation contributed to the decreased LA contractility (19). After Cox maze III surgery for paroxysmal AF, Lönnerholm et al. (20) demonstrated a decrease in the maximal left and right atrial areas after six months with a return to preoperative values at 56 months, but the left and right atrial contractility was significantly reduced after both six and 56 months versus baseline. The initial reduction in the atrial areas was proposed to depend on atrial scarring due to extensive surgery, while the later deterioration of atrial dimensions and function in patients with SR was unexpected.

Consistent with our approach, Tops et al. (21) compared patients in SR before and 14 months after percutaneous catheter ablation of AF and found significant improvement in the LV longitudinal strain and strain rate without a change in the LVEF, while significant deterioration was seen in patients remaining in AF. This also gives some support to the findings in our study, that patients in SR at surgery and follow-up, although they did not show distinct improvement, showed no or less deterioration than patients in AF at both time-points.

In another study by Boyd et al, patients with chronic AF had significant persistent left atrial dysfunction, despite restoration and maintenance of SR, six months after intraoperative linear radiofrequency ablation (22). Global and regional atrial dysfunction was present in ablated patients as compared to patients undergoing DC cardioversion only. In the present study, we allowed both patients with paroxysmal and persistent/permanent AF to participate. However, consistent with the findings of Boyd et al (22), we found a significant difference in the decrease of the mitral flow velocity during atrial systole between the cryoablated patients and control patients who were in SR at surgery and at follow-up, implying that the ablation procedure itself was at least in part responsible for some deterioration, found as late as 22 months after surgery. The exclusion of the atrial appendage in ablated patients might possibly have contributed.

The duration and possibly the type of AF, whether or not symptomatic, is no doubt of importance and, if long, considerable structural remodeling may have taken place to make reversal less probable. In addition, patients undergoing concomitant intraoperative ablation have considerable structural heart disease, constituting a sicker subpopulation that is likely to be more difficult to treat than patients with primary electrical disease. In our study, a history of myocardial infarction in 69% of the cryoablated and 46% of the control patients may have added to the risk of a deterioration of the postoperative ventricular function and dimensions.

When applied endocardially, cryoablation carried a minimal risk of thrombus formation and preserved underlying tissue architecture, probably because of its preservation of collagen tissue. There seemed to be a low risk of adjacent tissue injury, e.g. the circumflex coronary artery and the esophagus, compared to RF energy (23). In an animal study with epicardial cryo clamp lesions on the beating heart, Milla et al. (24) demonstrated conduction block in pulmonary veins and the left atrial appendage even though full transmurality was not achieved. In the same study, linear epicardial cryoablation using a 10-cm linear cryo probe demonstrated transmurality in 84% of the applications. In our study, cryoablation was performed epicardially on a vacuum “emptied” beating heart, on-pump, to minimize the re-warming effect of intracardiac blood flow.

### Limitations

We used a case-control study design and cannot exclude that a randomized study would have generated slightly different results. However, this would most probably have needed a multicenter trial design, and standardized echocardiographic measurements would then have been more difficult to achieve. The numbers of patients in the subgroups are small, and the results, although statistically significant, thus should be regarded as hypothesis generating rather than definite answers.

## Conclusions

Patients undergoing CABG with or without concomitant left atrial epicardial cryoablation showed a pattern of decreased atrial and ventricular function at long-term follow-up. Differences were found between patients who were in SR or AF both at operation and follow-up, respectively, but the subgroups were small and the results should be interpreted with caution and need to be confirmed in a larger patient population. SR before surgery and the left atrial area at baseline were independently predictive of SR at long-term follow-up.
